# Developing and Validating an Intelligent Mouth-Opening Training Device: A New Solution for Restricted Mouth Opening

**DOI:** 10.3390/s24061988

**Published:** 2024-03-20

**Authors:** Hao Wu, Zilin Wang, Jing Han, Tianchi Wu, Guangtao Zhai, Chenping Zhang, Jiannan Liu

**Affiliations:** 1 School of Health Science and Engineering, University of Shanghai for Science and Technology, Shanghai 200093, China; 211410175@st.usst.edu.cn; 2 Department of Oral and Maxillofacial Head and Neck Oncology, Shanghai Ninth People’s Hospital, Shanghai Jiao Tong University School of Medicine, Shanghai 200011, China; 117073@sh9hospital.org.cn; 3 Department of Oral and Maxillofacial Surgery, School and Hospital of Stomatology, Jilin University, Changchun 130021, China; wzl071436@hotmail.com; 4 College of Stomatology, Shanghai Jiao Tong University, Shanghai 200011, China; 5 National Center for Stomatology, Shanghai 200011, China; 6 National Clinical Research Center for Oral Diseases, Shanghai 200011, China; 7 Shanghai Key Laboratory of Stomatology, Shanghai 200011, China; 8 Shanghai Research Institute of Stomatology, Shanghai 200011, China; 9 Department of Orthopaedics and Traumatology, The University of Hong Kong, Hong Kong SAR, China; wuuuhku@connect.hku.hk; 10 Institute of Image Communication and Network Engineering, Shanghai Jiao Tong University, Shanghai 200240, China; zhaiguangtao@sjtu.edu.cn; 11 School of Electronic Information and Electrical Engineering, Shanghai Jiao Tong University, Shanghai 200240, China

**Keywords:** trismus, mouth-opening exercise, the intelligent mouth-opening training device, remote guidance, quantification of exercises

## Abstract

Restricted mouth opening (trismus) is one of the most common complications following head and neck cancer treatment. Early initiation of mouth-opening exercises is crucial for preventing or minimizing trismus. Current methods for these exercises predominantly involve finger exercises and traditional mouth-opening training devices. Our research group successfully designed an intelligent mouth-opening training device (IMOTD) that addresses the limitations of traditional home training methods, including the inability to quantify mouth-opening exercises, a lack of guided training resulting in temporomandibular joint injuries, and poor training continuity leading to poor training effect. For this device, an interactive remote guidance mode is introduced to address these concerns. The device was designed with a focus on the safety and effectiveness of medical devices. The accuracy of the training data was verified through piezoelectric sensor calibration. Through mechanical analysis, the stress points of the structure were identified, and finite element analysis of the connecting rod and the occlusal plate connection structure was conducted to ensure the safety of the device. The findings support the effectiveness of the intelligent device in rehabilitation through preclinical experiments when compared with conventional mouth-opening training methods. This intelligent device facilitates the quantification and visualization of mouth-opening training indicators, ensuring both the comfort and safety of the training process. Additionally, it enables remote supervision and guidance for patient training, thereby enhancing patient compliance and ultimately ensuring the effectiveness of mouth-opening exercises.

## 1. Introduction

The treatment of oral and maxillofacial malignant tumors involves comprehensive approaches, with surgery serving as the primary intervention [1,2,3,4,5]. However, tumor destruction and surgical resection result in a loss of tissue structure. Post-operative scarring and radiotherapy-induced fibrosis can lead to temporomandibular joint (TMJ) sclerosis and masticatory muscle fibrosis. Additionally, due to psychological fear of pain, patients might limit the range of motion of their mandible. Ultimately, these factors lead to restricted mouth opening (RMO) [6,7,8,9,10,11], subsequently resulting in malnutrition or even cachexia, posing a serious threat to patients’ lives [12,13,14]. RMO has emerged as one of the most common complications following treatment for head and neck malignant tumors, with a reported prevalence of trismus ranging from 8% to 62% [15,16]. For patients with head and neck cancer, the incidence of RMO is related to the tumor location, tumor destruction structure, surgical scar, and radiotherapy fibrosis.

However, there is currently a lack of consensus among experts regarding the treatment of RMO. Methods to alleviate RMO include medication, modified radiotherapy techniques, or surgical intervention. Surgical intervention may be necessary for patients with severe or prolonged RMO [17], but surgical removal of some tissue structures may cause other complications. Therefore, the current strategy focuses on prevention, preventing the deterioration of RMO, and restoring oral function through conservative treatment as much as possible.

Research has shown that mouth-opening exercises are an effective means of preventing the occurrence of trismus and reducing its severity [18]. The existing methods for mouth-opening exercises include both active and passive jaw movement exercises. Passive exercises involving jawbone movements have been widely acknowledged as effective rehabilitation techniques, encompassing guided finger training methods, wedge occlusal pad training methods, and traditional mouth-opening trainers. Regardless of the method used, the effectiveness of mouth-opening exercises largely depends on the patient’s compliance [17]. Additionally, during the patient’s home training, incorrect utilization of the mouth-opening device or the use of sudden force may result in TMJ injury, consequently exacerbating the degree of RMO. Consequently, the primary focus in the management of trismus has shifted toward the development of a safe and effective mouth-opening training device, accompanied by scientifically and systematically designed training methods.

Our research group successfully designed an intelligent mouth-opening training device (IMOTD) that elevates basic mechanical mouth exercises into an interactive rehabilitation experience involving the ‘doctor–patient–family’ relationship. This device operates on a three-in-one principle encompassing ‘mouth-opening training, intelligent schemes, and cloud-based platforms’. Patients can benefit from remote online guidance and supervision of their mouth-opening exercises, not only enhancing patient compliance but also augmenting the effectiveness of the exercise.

This article primarily introduces an innovative mouth-opening system that utilizes electronic sensors and a cloud platform to monitor patients’ exercise compliance, examining it from the perspectives of mechanical design, piezoelectric sensor (Wuyi Jiachen Electronic Technology Co., Ltd., Jinhua, Zhejiang province, China) calibration validation, and finite element analysis. The rehabilitation effects are validated against those obtained through clinical practices; moreover, we discuss the improvements and advantages of the proposed approach compared with purely mechanical devices.

## 2. Design of IMOTD

### 2.1. Design Concept

The design concept of the IMOTD revolves around addressing the needs of patients with limited mouth opening as the core focus. The design adheres to ergonomic principles, utilizes safe and non-toxic materials, and complies with relevant regulations and standards. The aim is to provide a scientific and humanized rehabilitation assistant tool. The device is suitable for patients with limited mouth opening, serving not only those recovering from head and neck cancer treatment but also individuals affected by other factors leading to limited mouth opening.

The primary function of the mouth-opening device is to open the patient’s upper and lower jaws gradually and steadily, preventing potential joint damage that could arise from sudden speed changes in traditional methods. Consequently, in the device’s structural design, a multi-level limit protection mechanism is essential to adapt to the mouth-opening training of different mouth sizes. The limit screw can adjust the range of mouth opening, gradually setting different degrees of opening according to various stages of exercise tasks to prevent excessive force from injuring the TMJ.

During the oral opening movement in the human body, the mandible undergoes both rotational and sliding motions. When the mandible transitions from the closed movement to the open state, the muscles responsible for opening contract while those for closing relax, enabling the mandible to move downward and backward. To optimize the opening effect, the internal sliding mechanism of the mouth opener needs to follow a specific trajectory. Considering structural processing and manufacturing costs, the motion path of the sliding mechanism in the mouth-opening device is designed to be a circular arc.

**Figure 1:**
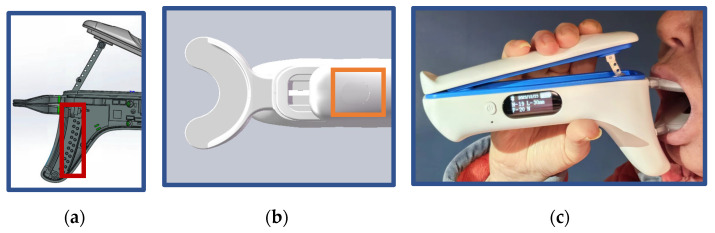
(**a**) The location of the infrared sensor shown in the red square in the figure can accurately sense the change in mouth opening; (**b**) the location of the piezoelectric sensor shown in the yellow square in the figure; and (**c**) a photo demonstrating clinical usage of the IMOTD.

To quantitatively and precisely monitor mouth-opening exercises for patients, the mouth-opening device utilizes an infrared sensor (highlighted in red in Figure 1a) and a piezoelectric sensor (highlighted in yellow in Figure 1b) to record both the force exerted and the amplitude of movement during each mouth-opening movement. Considering that the normal maximum interincisal opening (MIO) for healthy adults ranges between 37 and 45 mm, the upper and lower occlusal plate stroke displays were set within the range of 0–50 mm. To achieve this, the infrared sensor array was designed with receivers placed at 5 mm intervals, covering the full spectrum from 0 to 50 mm. Additionally, given that the normal adult anterior teeth bite force falls between 270 and 290 N, the piezoelectric sensor’s measurement range was configured from 0 to 300 N.

The IMOTD features a display screen on its side that presents the mouth-opening degree, resistant forces, the number of mouth-opening exercises, Bluetooth connectivity, and power status (Figure 1c). This screen allows family members to observe the patient’s training effect during home exercises and provide timely reminders. Simultaneously, the device’s internal Bluetooth connects to the patient’s mobile phone WeChat applet, enabling automatic data upload during exercises. The WeChat applet displays data and uploads these data to the cloud. Through the WeChat applet’s doctor portal, doctors can access and review the patient’s training data, providing remote guidance and supervision, thereby achieving functions like data visualization and remote training oversight.

### 2.2. Mechanical Perspective

**Figure 2:**
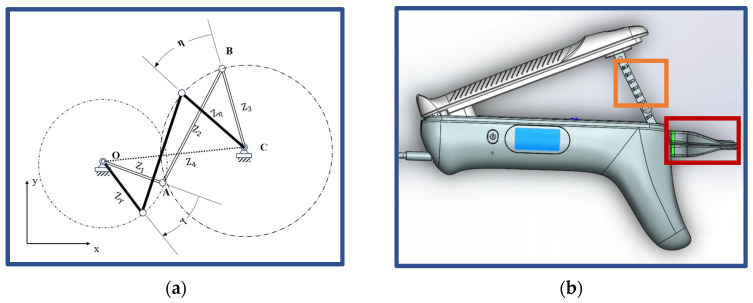
(**a**) Schematics of the four-bar linkage concept, where $\vec{CB}$ represents the handle of the mouth-opening device and $\vec{OA}$ represents the lower jaw of patients; (**b**) a pattern diagram of the IMOTD, where yellow box highlighting the stop holes on the metal beam and red box highlighting occlusal plates.

The mechanical structures were designed to ensure joint safety during the mouth-opening exercises, as depicted in Figure 2.

The four-bar linkage system operates by pressing the input bar $\vec{CB}$ to generate a specific motion on the output bar $\vec{OA}$ representing the lower jaw of the patient. The velocity and acceleration of the output bar are determined by the device configuration, and patient-specific output properties can be achieved by modifying the size of the input and output bars. For patients of various ages and anatomical features, an optimal device ensures a smooth rehabilitation exercise, maintaining a ratio of output over input equal to or below 1. Moreover, once pressure on the input bar is released, there is no residual force on the lower jaw of the patient, preventing over-extension of the mouth (Figure 2a).

The body of the IMOTD is made of Acrylonitrile Butadiene Styrene (ABS), ensuring a lightweight construction for easy portability. The slider connected to the occlusal plate is made of 7075 aluminum alloy, which increases the strength of the structure to resist the bite force.

Stop holes, distributed with a small spacing of 5 mm on the coupler bar ($\vec{AB}$ in Figure 2a, yellow-highlighted in Figure 2b), control the extent of mouth distraction for patients, enabling them to perform exercises slowly and steadily.

Metal beams, fabricated from 7075 aluminum alloy (highlighted in yellow in Figure 2b), were integrated into the handles to augment the stiffness of the device, ensuring zero deformation of the structure during regular rehabilitation exercises.

The occlusal plate (highlighted in red in Figure 2b), made of polycarbonate (PC), is relatively lightweight and thin, facilitating ease for patients to pull out and insert the occlusal plate.

### 2.3. Intelligent Monitoring

**Figure 3:**
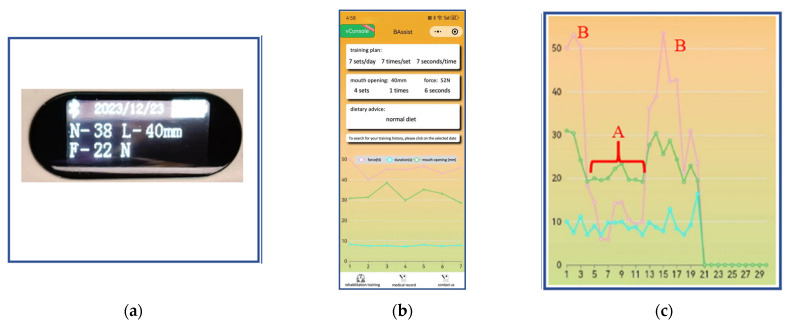
(**a**) Display screen: N is the cycles of mouth-opening exercises, L is the mouth-opening degree, and F is resistance forces; (**b**) WeChat applet patient side: training plan, mouth opening, force, dietary advice, and training effect diagram. (**c**) The above illustration shows an example displaying the exercise data for 21 days. The pink line represents the resistant forces, the green line represents the average distraction distance, and the light blue line represents the duration of mouth opening (the horizontal axis is the number of exercising days). Bracket A illustrates a terrible compliance, while peak B demonstrates the good practice.

After patients log into the WeChat app (Shenzhen Tencent Computer System Co., Ltd., Shenzhen, Guangdong province, China), they can input treatment-related data and complete the adjustment questionnaire (Gothenburg Trismus Questionnaire). During training, the display screen shows the mouth-opening degree, resistance forces, and the cycles of mouth-opening exercises completed (Figure 3a). Family members can monitor patient training through display data. Based on the collected mouth-opening degree, dietary recommendations are provided, and a training program is displayed (Figure 3b). The training data are automatically uploaded to the cloud, allowing access for doctors through the application’s doctor interface to view the patient’s training effect diagram (Figure 3c).

Piezoelectric sensors (Miniature force sensor 700) are mounted at one end of the coupler bar. The functionality of the Miniature Force Sensor 700 lies in its ability to transform mechanical forces exerted upon it into electrical signals through the utilization of the piezoelectric effect. This mechanism facilitates the attainment of exceptionally precise measurements for resistant forces. The sensor is adapted to record crucial parameters such as the maximum resistant forces of the mouth, duration time of maximum resistant forces, and mouth-opening degree. Moreover, a buzzer rings when the duration of the maximum resistance force lasts 10 s. After completing the daily rehabilitation exercise (consisting of seven groups of mouth-opening exercises), the average resistance, average distraction distance, and average duration of maximum forces for that day are presented in a line chart (Figure 3c).

If a patient cannot maintain the exercise at the desired level (as indicated in segment A of Figure 3c), clinicians can promptly intervene to remind the patient of effective exercise levels. Segment B in Figure 3b informs clinicians about physical changes occurring in patients. In comparison with segment A, a similar mouth-opening extent in segment B is associated with approximately double the resistant forces, suggesting a potential increase in the stiffness of soft tissues surrounding the mouth. This signal may prompt clinicians to modify the patient’s rehabilitation exercise to better control the fibrosis transition.

## 3. Validation Methods

### 3.1. Piezoelectric Sensor Calibration

**Figure 4:**
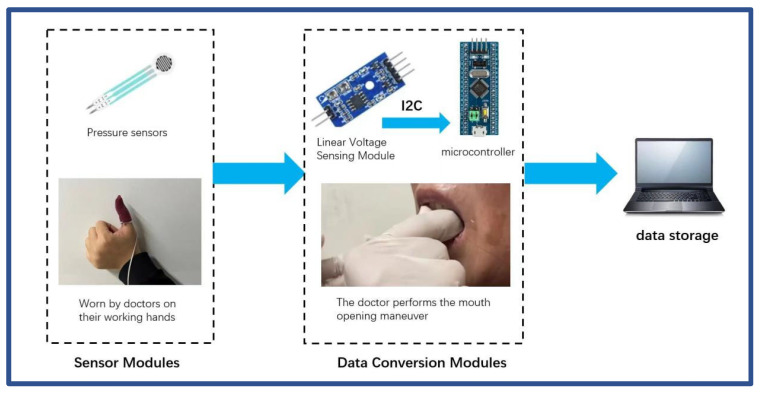
The force measurement process of the doctor’s finger with an FSR thin-film pressure sensor.

When the IMOTD is turned on, the calibration process of the piezoelectric sensor involves standardization to zero based on the initial value obtained from the sensor when the device is turned on. In addition, as the sensor is not mounted at the nearest end next to the patient’s mouth, the sensor measuring the maximum resistant force was validated against mouth-opening exercises using fingers. To assess the accuracy and stability of the intelligent device’s output force, data were collected on the output force from both the doctor’s finger-mouth-opening exercise and the IMOTD. The endpoint considered was the same degree of pain in the TMJ area of the same patient. The doctor’s finger detection method was conducted as follows: The FSR thin-film pressure sensor (Jiangsu Changxian photoelectric technology Co., Ltd., Yangzhou, Jiangsu province, China) is placed in the area of the doctor’s finger force to ensure stable contact between the sensor and the tooth. The resistant force measured with the FSR sensor is recorded by the data acquisition system, ensuring real-time data collection during the mouth-opening process (data sampling frequency, 10 Hz) (Figure 4). Meanwhile, the IMOTD was utilized by the patient under the guidance of a doctor, and the strength data output by the IMOTD was recorded with the data acquisition system. Both test methods involve the collection of data 12 times to ensure reliable results and averages.

Test Indicators: (1) Comparison of Average Output Force: Calculate the average output force of both the doctor’s finger-mouth-opening training and the IMOTD. Comparing their average output forces, if the intelligent device’s average output force closely resembles that of the doctor’s finger training, it can be considered an effective replication of the doctor’s technique in strength. (2) Analysis of Standard Deviation and Coefficient of Variation: Calculate the standard deviation of the output force of the doctor’s finger mouth-opening technique and the IMOTD. Comparing the standard deviation of the two, if the standard deviation of the output force of the IMOTD is similar to that of the doctor’s finger mouth-opening technique, it shows that the mouth-opening device can restore the stability of the doctor’s technique in terms of strength. (3) Correlation Analysis: Conduct a correlation analysis between the output force of the doctor’s finger-mouth-opening exercise and that of the IMOTD. Calculate correlation coefficients, like the Pearson correlation coefficient, to gauge the linear relationship between the two. A correlation coefficient near 1 signifies a strong linear relationship between the output force of the IMOTD and the output force of the doctor’s finger mouth-opening exercise.

### 3.2. Finite Element Analysis

#### 3.2.1. In Silico Safety Inspection

Stop holes on the coupler beam (highlighted in yellow in Figure 2b) are positioned off the midline, leading to stress concentration on the side with a smaller hole-to-edge distance. Consequently, a finite element (FE) model of the coupler beam was developed to assess the structure’s load-bearing capacity. Geometry partitions and refined meshes were mapped, and a hard contact with a friction coefficient of 0.05 was applied to simulate the interaction between stop holes and rigid nails. Material properties, mesh sensitivity checks, and the Mises stress distribution are provided in the Supplementary Materials.

Based on clinical tests, the maximum resistant forces of patients with limited mouth opening were determined, and a high safety factor of 4 was assigned. Finally, the compression force for the loading case was defined. The FE analysis assesses whether the maximum Mises stress in the metal connecting rod structure is below the material yield strength under the final concentrated force load, thereby evaluating the safety of the metal connecting rod structure.

#### 3.2.2. Occlusion Plate Insertion Method

**Figure 5:**
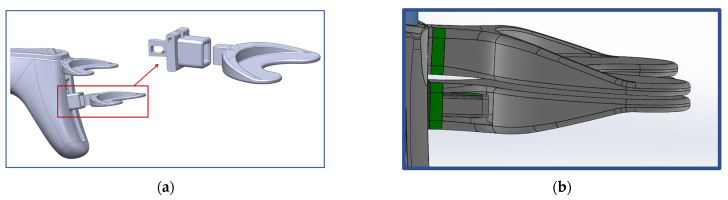
(**a**) The occlusal plate is designed to be inserted into the slider. (**b**) The slider is designed to be inserted into the occlusal plate.

Prior to activating the IMOTD, the patient must initially connect the occlusal plate to the slider of the device. The occlusal plate is made of polycarbonate, and the slider is made of a 7075 aluminum alloy. The design approach takes into consideration the maximum resistant forces of mouth opening and the insertion force required when installing the occlusal plate. The connection between the occlusal plate and the slider involves two distinct methods. The first method consists of the occlusal plate being inserted into the slider (Figure 5a), which is designed as an interference fit. The second method consists of the slider being designed to be inserted into the occlusal plate (Figure 5b), where both have matching contact surface sizes without exerting extrusion force. Through conducting finite element analyses on various design types and sizes, we calculated both the insertion force of the occlusal plate and the maximum resistant forces.

### 3.3. Pilot Study

#### 3.3.1. Patient Preparation

**Figure 6:**
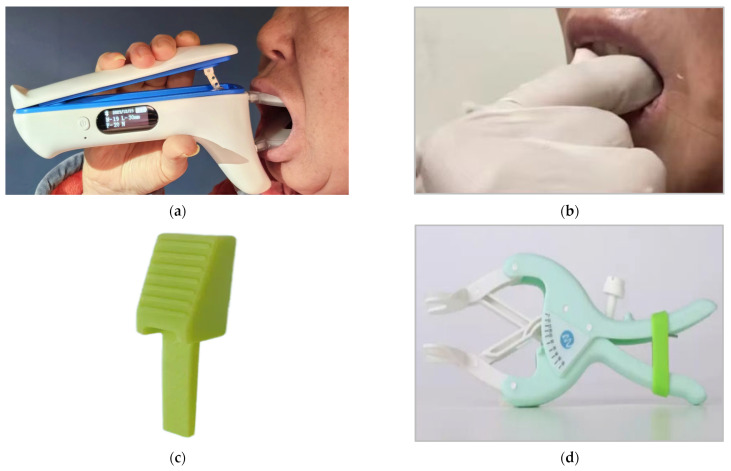
(**a**) The IMOTD used by patients 1, 2, and 3; (**b**) the finger training method used by patient 4; (**c**) the wedge occlusal pad used by patient 4; and (**d**) the traditional mouth-opening device used by patient 5.

Patients were routinely instructed to open their mouths after surgery. Typically, training programs include finger training methods, wedge occlusal pad training methods, and traditional mouth-opening trainers. To evaluate the training effects of these three different mouth-opening methods, five patients were included and consented to enrollment in a clinical trial at a single center. All patients in this study were diagnosed with buccal cancer, and the surgical approach involved a combined operation on the buccal, mandible, and neck, with reconstruction utilizing the anterolateral thigh (ALT) flap. There were no restrictions on mouth opening pre-operatively, and the oral cavity wounds exhibited complete healing within three weeks post-operation. Mouth-opening exercises commenced for all patients in the third week post-operation. Patients 1, 2, and 3 used the IMOTD for training (Figure 6a); patient 4 complained of finger pain due to prolonged finger training and was alternately trained with a wedged silicone occlusal pad (Figure 6b,c); and patient 5 used a traditional mouth-opening device (Figure 6d). All patients began training 3 weeks after surgery. At this time, the patients’ wounds had healed, mitigating their fear of pain or wound dehiscence. Following a standardized radiotherapy protocol, radiotherapy was initiated four weeks after the operation. All patients underwent radiotherapy 30 times, and the changes in the MIO distance before radiotherapy are presented in Table 1. The study protocol was approved by the Medical Ethics Committee of the Ninth People’s Hospital Affiliated with Shanghai Jiao Tong University School of Medicine (SH9H-2022-T305-1).

#### 3.3.2. Exercise Protocol

For patients 1, 2, and 3 undergoing rehabilitation with the intelligent mouth-opening training system, they were instructed to follow the 7-7-7 protocol, which consisted of seven sessions per day, seven openings/closings per session, and a seven-second stretch for each opening. On the other hand, patients 4 and 5 were instructed to keep their mouths open for 1 min at a time, relax for 30 s, and repeat the operation for 30 min as a group. They were encouraged to practice at least 2–3 exercise groups every day.

#### 3.3.3. Follow-Up Time

Follow-up evaluations were performed at 4 weeks after surgery, the 5th radiotherapy session, the 10th radiotherapy session, the 20th radiotherapy session, the 30th radiotherapy session, 1 month after radiotherapy, 3 months after radiotherapy, 5 months after radiotherapy, and 7 months after radiotherapy. Notably, patients 2 and 3 continued their training regimen for less than 7 months after radiotherapy.

## 4. Validation Results

### 4.1. Piezoelectric Sensor Calibration

Based on the average comparison, the average output force of the doctor’s finger mouth-opening technique (the FSR thin-film pressure sensor) was 45 N, whereas the average output force of piezoelectric sensors in the IMOTD was 34 N. Although the average output force of the smart mouth opener was slightly lower than that of the doctor’s technique, the gap between the two was not significant.

The comparison of standard deviations indicates that the doctor’s finger-mouth-opening technique (the FSR thin-film pressure sensor) had a standard deviation of 14 N, whereas the output force of the IMOTD had a standard deviation of 12 N. This suggests that the smart mouth opener more effectively replicates the stability of the doctor’s technique in terms of strength. Additionally, the close proximity of the standard deviations indicates well-calibrated piezoelectric sensors within the smart mouth device.

**Figure 7:**
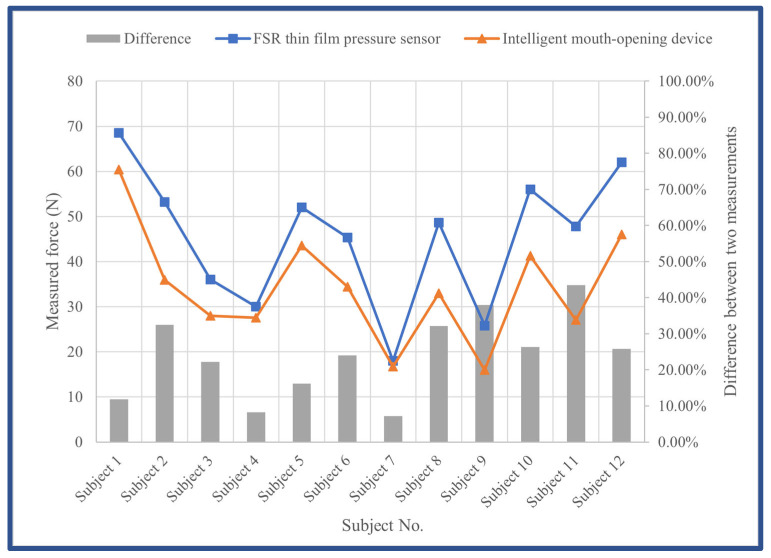
Maximum resistant forces were measured using both the IMOTD and the commercial pressure sensor (FSR thin-film pressure sensor).

The FSR thin-film pressure sensor consistently yielded higher measurements, exhibiting an average difference of 24% (Figure 7). A Pearson correlation analysis was conducted, revealing a robust linear relationship between the forces measured for the IMOTD and the commercial pressure sensor. The Pearson correlation coefficient was 0.923 at the 0.01 significance level (two-tailed significance test), indicating a strong correlation and excluding random variation.

### 4.2. Finite Element Analysis

#### 4.2.1. In Silico Safety Inspection

**Figure 8:**
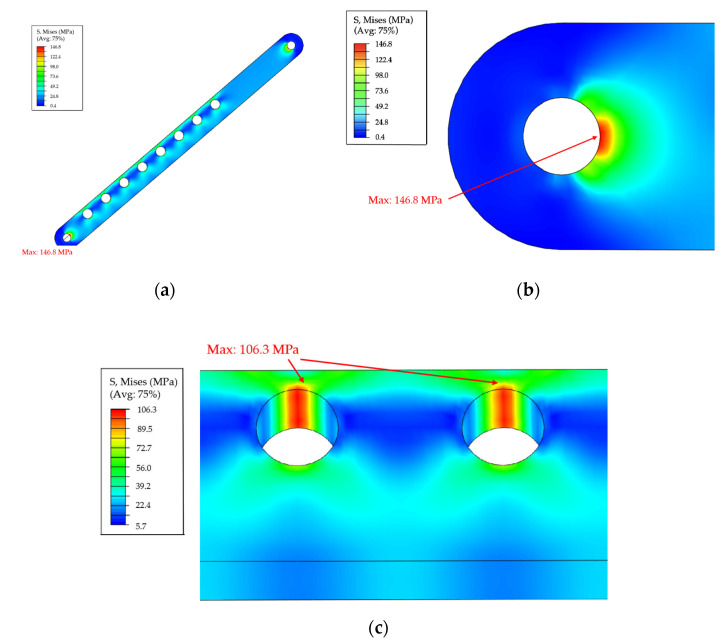
Mises stress distribution under a 280 N compression (the stop nail and metal pins are hidden): (**a**) coupler bar; (**b**) pinhole; and (**c**) stop holes.

Based on limited clinical records, the maximum resistant forces observed in patients with trismus ranged from 18 N to approximately 70 N. With a high safety factor of 4, the ultimate loading case was set to a compression of 280 N. Finite Element (FE) results demonstrated that, under 280 N compression, the coupler beam exhibited a maximum Mises stress of up to 146.8 MPa (Figure 8c) around the pinholes at both ends, indicating that the structure maintains a high level of safety. Despite the stop holes being designed off-midline of the beam, the Mises stress pattern showed limited influence around the joint holes (Figure 8a). The stress concentration around the stop holes (106.3 MPa) was significantly smaller than the material yield strength (Figure 8b).

#### 4.2.2. Optimization of Insertion Force

Various Finite Element (FE) models were generated to calculate the insertion force, utilizing a friction coefficient of 0.20. Different geometrical configurations were compared to determine the appropriate interference fit (Table 2).

Type I and type II occlusal plates were examined: one with tapered extrusion from the base to the other end of the occlusal plate, and another with 4 mm straight extrusion and 3 mm tapered extrusion. Inappropriate interference can result in either a large or small insertion force, with an ideal insertion force being around 300 N. Comparing configuration 5 and configuration 7, even though the insertion forces are approximately the same, configuration 7 exhibited less stress concentration (von Mises stress of 49.53 MPa, which is smaller than the yield strength of the PC material).

Type III occlusal plates were also examined: as the tensile strength of the PC material is 62 MPa, the inner size of the occlusal plate is consistent with the outer size of the slider, and there is no mutual extrusion on either side. Without considering the tensile stress caused by the assembly, the maximum resistance is 154.8 N. According to clinical records, the maximum resistance observed in patients with RMO is about 70 N, and the safety factor of the type III occlusal plate is lower than that of type I and type II occlusal plates.

As the simulation was based on a very ideal situation, if the material’s performance cannot reach 62 MPa, the actual interference fit may create prestress in the occlusal plate, and the prestress is tensile stress. If this stress reaches 62 MPa, it could lead to damage. If some tensile stress is generated post-production in the type III occlusal plate, the maximum resistance is less than 154.8 N. However, the resistance of configuration 7 is 210.9 N. Therefore, configuration 7 of type II appeared to be the most favorable option under these circumstances.

### 4.3. Pilot Study

#### 4.3.1. The Results of IMOTD Use

At three weeks after surgery, patient 1 had an MIO of 20 mm. Before radiotherapy (4 weeks after surgery), patient 1 could open their mouth to 30 mm. The MIO remained at 28 mm after 10 radiotherapy sessions, 28 mm after 20 sessions, and 27 mm after 30 sessions. Consistent rehabilitation efforts helped patient 1 maintain a controlled, restricted mouth opening, achieving a 30 mm MIO after one month. However, during the 4th and 5th months after radiotherapy, patient 1 suspended training for two months due to work reasons but resumed afterward. The MIO was 30 mm at 7 months after radiotherapy. For patient 2, the MIO was 15 mm at three weeks after surgery, and the MIO was 25 mm at 4 weeks after the operation. Patient 2 experienced severe mucosal reactions during radiotherapy. Due to pain, the patient was hesitant to open their mouth, reaching 20 mm after 20 radiotherapy sessions. Subsequently, the follow-up situation stabilized, with MIO further increasing to 25 mm and remaining stable. In contrast, patient 3 maintained the same MIO of 35 mm throughout the observation period.

**Figure 9:**
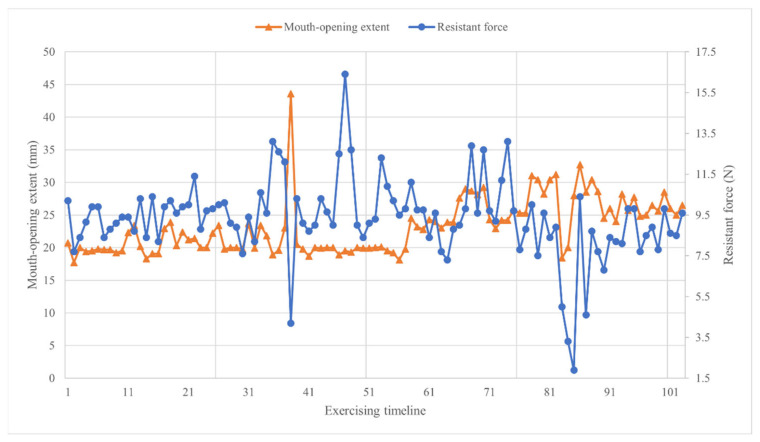
Patient 1 exercising records (triangle dots represent the mouth-opening extent and circle dots represent the resistant force).

Significantly, after one week of mouth-opening exercises, the MIO measurements for both patient 1 and patient 2 exceeded the post-operative values at 4 weeks, indicating positive progress in mouth-opening functionality. Figure 9 illustrates the exercise records for patient 1, revealing an increase in mouth-opening extent after approximately three months of exercises, while the resistant force remained at the same level. These trends suggest that the muscles surrounding the mouth underwent rehabilitation toward a more relaxed state, highlighting the effectiveness of the mouth-opening exercises.

#### 4.3.2. Comparison with the Traditional Open-Mouth Training Method

**Figure 10:**
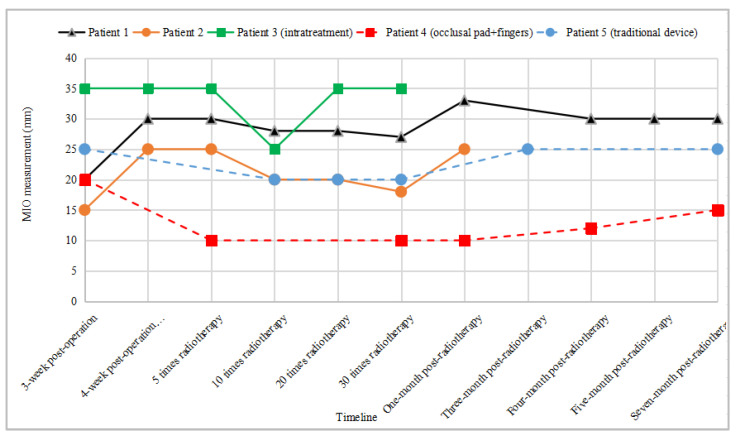
MIO trends of five patients against the treatment timeline (patients 2 and 3 are still in training).

After 9 months of training, the effects of different mouth-opening exercise methods were observed. Before the start of training, the MIO of patient 2 was less than that of patients 1 and 4, while patient 5 had the largest mouth-opening extent. After a period of training, the MIO of patients 1 and 2 increased the most. As depicted in Figure 10, the increase in mouth-opening amplitude for patient 1 (black triangle data points) and patient 2 (orange square data points) is more significant than that for patient 4 (red square data points) and patient 5 (blue circle data points). Meanwhile, patient 3 maintained the same MIO after 30 sessions of radiotherapy (green circle data points). Patient 2 (10th session of radiotherapy), patient 3 (30th session of radiotherapy), patient 4 (5th session of radiotherapy), and patient 5 (10th session of radiotherapy) refrained from opening their mouths due to difficulty tolerating a sore throat. Throughout the radiotherapy period, changes in the patients’ mouth-opening extent were also influenced by radiotherapy mucosal reactions, pain, and acute reactions. All patients in this study had the same tumor location, surgical resection range, and radiotherapy dose and range. Even if patients experience different mucosal reactions or pain reactions during radiotherapy, leading to significant changes in mouth opening within a short period, once acute inflammation is controlled (usually within 1 week), doctors still encourage them to continue mouth-opening training. Therefore, the mouth-opening training effect of these five patients can directly reflect the different effects of various mouth-opening training methods.

Patients using the IMOTD received reminders through the app if they deviated from the training schedule. This served as both supervision and a reminder, contributing to a more effective recovery of mouth opening. Regular follow-ups were conducted for patient 4 and patient 5, during which the doctor emphasized the importance of consistent mouth-opening exercises. The success of the patients’ daily training at home relied on their individual awareness and commitment. Therefore, the effectiveness of mouth-opening training depends not only on the method of mouth-opening training but also on patients’ compliance. The IMOTD can significantly improve patient compliance.

## 5. Discussion

### 5.1. Comparison with Other Mouth-Opening Devices

Existing studies focused on the Therabite^®^, EZbite^®^, and Dynasplint System^®^ have consistently shown positive outcomes in mouth-opening rehabilitation exercises for patients with trismus [19,20,21,22]. However, few devices have been designed to enhance patient compliance and assist rehabilitation. In comparison with traditional oral rehabilitation devices, the proposed intelligent mouth-opening training system enhances patient compliance, consequently improving the effectiveness of rehabilitation exercises. Despite the current limitations in clinical sample size due to clinical involvement rules, we believe there is a high possibility that the proposed device could effectively facilitate jaw movement exercises in the control and management of trismus.

#### 5.1.1. Safety and Convenience

The IMOTD is specifically designed for patients with a mouth opening greater than 5 mm, providing a more versatile solution compared with traditional mouth-opening devices that often require a certain degree of mouth opening before use; for example, the mouth-opening device shown in Figure 6d requires a degree of mouth opening greater than 15 mm to be used. Traditional devices, often constructed with plastic (PC or ABS) structures, may suffer from plastic deformation after repeated use, leading to a reduction in mouth-opening extent and potentially providing clinicians and patients with inaccurate indicators. In contrast, the rigid structure of the proposed device not only ensures durability but also enhances patient comfort, eliminating the finger pain associated with manual or traditional gripping-method mouth-opening devices.

Furthermore, the proposed device features an ergonomic design, allowing users to hold it within the palm of their hand, providing easy and subjective control of compression forces. Upon releasing the handle, no additional compression is applied to the lower jaw, facilitating a quick restoration to its natural position and relieving pain and soreness [19]. In contrast, an alternative mouth-opening device, as depicted in Figure 6d, requires users to twist the handle on one side to enlarge the opening extent, and the difficulty of twisting the handle might inadvertently move the device, potentially causing secondary injury to the mandibular joint [23].

#### 5.1.2. Improvement in Compliance

The proposed system comprises both the IMOTD and software components. Patients utilize the IMOTD for their training sessions, and the data collected during these sessions is automatically uploaded to the cloud. This integration provides a platform for doctors to remotely monitor and assess the progress of their patients’ training. Accessible through a dedicated mobile application, doctors can review the data and make necessary adjustments to the training regimen. This remote supervision ensures compliance and minimizes subjectivity in patient reports. Research has indicated that maintaining consistency in mouth-opening exercise within the first six to nine months post-treatment is crucial to prevent irreversible changes due to scar formation and fibrosis [24]. Therefore, initiating mouth-opening exercises as early as possible and maintaining them consistently for 6–9 months after the operation can potentially prevent the occurrence of mouth-opening limitations. Patient 1 achieved favorable exercise results with a mouth opening of 20 mm before training and 30 mm after 7 months of training, possibly attributed to good compliance. In contrast, patient 5 exhibited poor compliance, resulting in a decrease in rehabilitation outcomes from 20 mm before exercise to 15 mm after 7 months of training. Patient 3, on the other hand, maintained the same MIO after 30 sessions of radiotherapy, resulting in consistent rehabilitation outcomes with a mouth opening of 35 mm before and 35 mm after 6 weeks of training. However, as the radiotherapy for patient 3 has concluded, their inflammatory response is expected to gradually subside. With adherence to regular exercise, the MIO of patient 3 still shows an increasing trend. A 35 mm mouth opening is considered normal and does not indicate a limitation that would impact the patient’s ability to eat various types of food [12,25,26].

#### 5.1.3. Quantitative Indicators

The quantitative aspects of this system are particularly significant. Specifying training durations and intensities enhances the effectiveness of rehabilitation and reduces the risk of joint damage resulting from prolonged mouth opening. As there is no standardized training program or expert consensus on the diagnosis and treatment of RMO. Therefore, doctors supervise the patient’s training data and design different training methods to upload to the small program according to the changes in training intensity that patients can tolerate. Patients who have undergone bone reconstruction may require targeted rehabilitation solutions to facilitate muscle reattachment and prevent reluctance to exercise, which can lead to restricted mouth opening. Moreover, the system stimulates the opening of the mouth’s pterygoid muscles, providing a comprehensive approach to rehabilitation.

Without specific metrics, patient compliance may result in misleading information on the underlying relationships. Grandi et al. have conducted a comparison of different exercises to determine their efficacy. Although no significant difference was found between the examined exercises, the lack of supervision from clinicians introduced uncertainty into the comparison [27]. The causes of trismus vary individually, making it essential to identify a tailored exercise pattern for patients with different extents of muscle fibrosis and various conditions of the temporomandibular joint.

In clinical routine, the passive mouth-opening extent is commonly regarded as an indicator of trismus recovery. However, some researchers argue that participants’ active mouth-opening extent and long-term mouth-opening extent may be more representative [23]. From our perspective, it is crucial to consider the maximum resistant force as well as to account for the extent of muscle fibrosis.

#### 5.1.4. Hygiene

In terms of hygiene, the intelligent mouth-opening training system ensures that patients can maintain a clean and sanitary practice, thereby reducing the risk of infection and addressing other oral health concerns. However, to maintain cleanliness during use, the occlusal pad needs frequent replacement, inevitably leading to increased usage costs.

### 5.2. Future Work

Looking ahead, future work on this system could involve the development of different-sized bite plates to accommodate various mouth widths, consider anterior dentition conditions, and prevent oral injuries during training. This adaptability and customization would further enhance the system’s efficacy and safety.

## 6. Conclusions

In summary, the introduction of the intelligent mouth-opening training system, which includes the IMOTD, represents a significant advancement in oral rehabilitation. It effectively addresses several limitations associated with traditional tools and techniques, provides quantifiable benefits, and enhances patient comfort, compliance, and overall oral health. The ongoing clinical pre-experiment preliminarily explored the effectiveness of the system’s training effects, showing significant improvements in training compliance. Future enhancements and customization options are likely to further enhance the system’s effectiveness and accessibility for a broader range of patients.

## 7. Patents

The intelligent mouth opening training device has applied for a Chinese invention patent (patent number: CN202011002311.3), a utility model patent (CN202022099425.6), and a design patent (CN202030578200.1), all of which have been authorized.

## Figures and Tables

**Table 1:** Basic information about the enrolled patients.

Patient No.	Gender	Age	Tumor Location	Treatment Regimens	Pre-Operative MIO * (mm)	Post-Operative MIO * (Mm) at 2 Weeks	Post-Operative MIO * (mm) at 3 Weeks	Post-Operative MIO * (mm) at 4 Weeks
1	M	38	Left buccal	S + R	40	20	20	30
2	M	70	Left buccal	S + R	35	15	15	25
3	F	68	Right buccal	S + R	42	35	35	35
4	M	71	Left buccal	S + R	27	20	20	20
5	M	68	Right buccal	S + R	35	25	25	25

M, male; F, female; MIO *, maximum interincisal opening; S + R, surgery plus radiotherapy.

**Table 2:** Insertion force of the occlusal plate when using various interference extents.

No.	Occlusal Plate (Outer Dimension)				Slider (Inner Dimension)			Insertion Force (N)
	Width (mm)	Height (mm)	Depth (mm)	Taper (degree)	Width (mm)	Height (mm)	Depth (mm)	
Type I The occlusal plate tapered from the base to the end 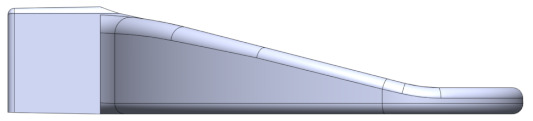								
1	5.8	8.2	7	1	5.7	8.1	8	1007.7
2	5.8	8.2	7	1	5.75	8.15	8	294.24
3	5.8	8.2	7	1	5.72	8.12	8	616.98
4	5.8	8.2	7	1	5.73	8.13	8	458.96
5	5.8	8.2	7	1	5.74	8.14	8	325.02
Type II straight cross-section followed by tapered to the end 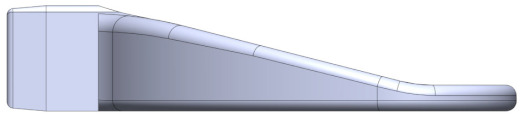								
6	5.8	8.2	3 (7)	1	5.74	8.14	8	1204.2
7	5.8	8.2	3 (7)	1	5.78	8.18	8	320.92
Type III straight cross-section 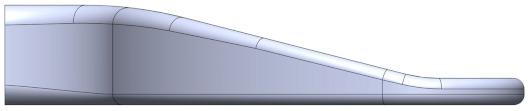 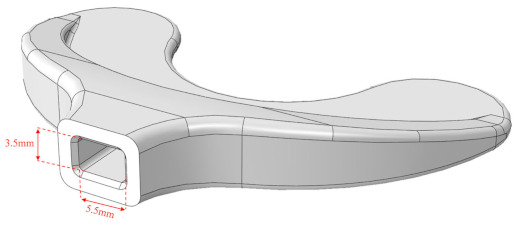								
8	5.5	3.5	7	0	5.5	3.5	8	-

Note: In type II, the depth parameter represents the depth of tapered extrusion; the value in parenthesis is the total length of tapered extrusion and straight extrusion.

## Data Availability

Data from this study are available from the corresponding author upon reasonable request.

## Supplementary Materials

The following supporting information can be downloaded at: https://www.mdpi.com/article/10.3390/s24061988/s1.

## Abbreviations

The following abbreviations are used in this manuscript:

TMJ	Temporomandibular Joint
RMO	Restricted Mouth Opening
IMOTD	Intelligent Mouth-Opening Training Device
ABS	Acrylonitrile Butadiene Styrene
PC	Polycarbonate
MIO	Maximum Interincisal Opening

## References

[1] Lee R., Slevin N., Musgrove B., Swindell R., Molassiotis A. (2012). Prediction of post-treatment trismus in head and neck cancer patients. Br. J. Oral Maxillofac. Surg..

[2] Bhrany A.D., Izzard M., Wood A.J., Futran N.D. (2009). Coronoidectomy for the Treatment of Trismus in Head and Neck Cancer Patients. Laryngoscope.

[3] Mardini S., Chang Y.-M., Tsai C.-Y., Coskunfirat O.K., Wei F.-C. (2006). Release and Free Flap Reconstruction for Trismus that Develops after Previous Intraoral Reconstruction. Plast. Reconstr. Surg..

[4] Huang I.Y., Wu C.F., Shen Y.S., Yang C.F., Shieh T.Y., Hsu H.J., Chen C.H., Chen C.M. (2008). Importance of patient’s cooperation in surgical treatment for oral submucous fibrosis. J. Oral Maxillofac. Surg..

[5] Mehrotra D., Pradhan R., Gupta S. (2009). Retrospective comparison of surgical treatment modalities in 100 patients with oral submucous fibrosis. Oral Surg. Oral Med. Oral Pathol. Oral Radiol. Endod..

[6] Bensadoun R.-J., Riesenbeck D., Lockhart P.B., Elting L.S., Spijkervet F.K.L., Brennan M.T. (2010). A systematic review of trismus induced by cancer therapies in head and neck cancer patients. Support. Care Cancer.

[7] Scherpenhuizen A., van Waes A.M.A., Janssen L.M., Van Cann E.M., Stegeman I. (2015). The effect of exercise therapy in head and neck cancer patients in the treatment of radiotherapy-induced trismus: A systematic review. Oral Oncol..

[8] Tveterås K., Kristensen S. (1986). The aetiology and pathogenesis of trismus. Clin. Otolaryngol. Allied Sci..

[9] Eipe N. (2005). The chewing of betel quid and oral submucous fibrosis and anesthesia. Anesth Analg..

[10] Johnson N.W., Warnakulasuriya S., Gupta P.C., Dimba E., Chindia M., Otoh E.C., Sankaranarayanan R., Califano J., Kowalski L. (2011). Global oral health inequalities in incidence and outcomes for oral cancer: Causes and solutions. Adv. Dent. Res..

[11] Bengtson B.P., Schusterman M.A., Baldwin B.J., Miller M.J., Reece G.P., Kroll S.S., Robb G.L., Goepfert H. (1993). Influence of prior radiotherapy on the development of postoperative complications and success of free tissue transfers in head and neck cancer reconstruction. Am. J. Surg..

[12] Dijkstra P.U., Kalk W.W.I., Roodenburg J.L.N. (2004). Trismus in head and neck oncology: A systematic review. Oral Oncol..

[13] Weber C., Dommerich S., Pau H.W., Kramp B. (2010). Limited mouth opening after primary therapy of head and neck cancer. Oral Maxillofac. Surg..

[14] Louise Kent M., Brennan M.T., Noll J.L., Fox P.C., Burri S.H., Hunter J.C., Lockhart P.B. (2008). Radiation-induced trismus in head and neck cancer patients. Support. Care Cancer..

[15] Johnson J., van As-Brooks C.J., Fagerberg-Mohlin B., Finizia C. (2010). Trismus in head and neck cancer patients in Sweden: Incidence and risk factors. Med. Sci. Monit..

[16] Watters A.L., Cope S., Keller M.N., Padilla M., Enciso R. (2019). Prevalence of trismus in patients with head and neck cancer: A systematic review with meta-analysis. Head Neck.

[17] Wu H., Zhou Z., Zhang C., Shen S., Liu J., Zhang C. (2021). The progress of post-treatment restricted mouth opening in oral and maxillofacial malignant tumor patients. Front. Oral Maxillofac. Med..

[18] Epstein J.B., Thariat J., Bensadoun R.J., Barasch A., Murphy B.A., Kolnick L., Popplewell L., Maghami E. (2012). Oral complications of cancer and cancer therapy. CA Cancer J. Clin..

[19] Li Y.H., Liu C.C., Chiang T.E., Chen Y.W. (2018). EZBite open-mouth device: A new treatment option for oral submucous fibrosis-related trismus. J. Dent. Sci..

[20] Pauli N., Johnson J., Finizia C., Andréll P. (2012). The incidence of trismus and long-term impact on health-related quality of life in patients with head and neck cancer. Acta Oncol..

[21] Dijkstra P.U., Huisman P.M., Roodenburg J.L.N. (2006). Criteria for trismus in head and neck oncology. Int. J. Oral Maxillofac. Surg..

[22] Stubblefield M.D., Manfield L., Riedel E.R. (2010). A preliminary report on the efficacy of a dynamic jaw opening device (dynasplint trismus system) as part of the multimodal treatment of trismus in patients with head and neck cancer. Arch. Phys. Med. Rehabil..

[23] Li Y.-H., Chang W.-C., Chiang T.-E., Lin C.-S., Chen Y.-W. (2018). Mouth-opening device as a treatment modality in trismus patients with head and neck cancer and oral submucous fibrosis: A prospective study. Clin. Oral Investig..

[24] Van der Geer S.J., van Rijn P.V., Kamstra J.I., Roodenburg J.L.N., Dijkstra P.U. (2018). Criterion for trismus in head and neck cancer patients: A verification study. Support. Care Cancer.

[25] Lee Y.-C., Wong T.-Y., Shieh S.-J., Lee J.-W. (2012). Trismus Release in Oral Cancer Patients. Ann. Plast. Surg..

[26] (1995). LENT SOMA tables. Radiother Oncol..

[27] Grandi G., Silva M.L., Streit C., Wagner J.C.B. (2007). A mobilization regimen to prevent mandibular hypomobility in irradiated patients: An analysis and comparison of two techniques. Med. Oral Patol. Oral Cir. Bucal.

