# Supplementary material for: Developing and Validating an Intelligent Mouth-Opening Training Device: A New Solution for Restricted Mouth Opening
Source: Sensors (Basel). 2024 Mar 20;24(6):1988. doi: 10.3390/s24061988 (PMC10975241; doi:10.3390/s24061988)
Supplement: Supplementary file 1 [file sensors-24-01988-s001.zip › sensors-2820956-supplementary.pdf]

Supplementary Materials:

To deliver the safety check of the coupler beam, a finite element (FE) model was established in Abaqus (SIMULIA, Dassault Systems, France), where a metal coupler beam with metal pins at both ends were constructed and threads in the stop holes of the limit screws were ignored. The Coulomb friction was set up between the metal pin and the metal coupler beam using the penalty method, with a friction coefficient of 0.05 due to the lubricant between the structures; hard contact (no penetration happening between the structures) was defined for the normal behavior of the contacting interface. The small sliding algorithm and surface-to-surface discretization method were implemented to rule out the mesh penetration between structures.

The coupler beam is fabricated by 7075 aluminum alloy , specific material properties of are listed in Table S1: .

Table S1: Material properties of 7075 aluminum alloy

	Unit	Value
Poisson's ratio	-	0.33
Young's modulus	GPa	70
0.2% yield strength	MPa	505
Shear strength	MPa	330

When the metal coupler beam was assembled with the metal pin, it was ensured that the position of the metal coupler beam aligned with the center of the metal pin to avoid additional bending moment because of eccentricity. The FE model is shown in Figure S1 with the dimensions of each component.

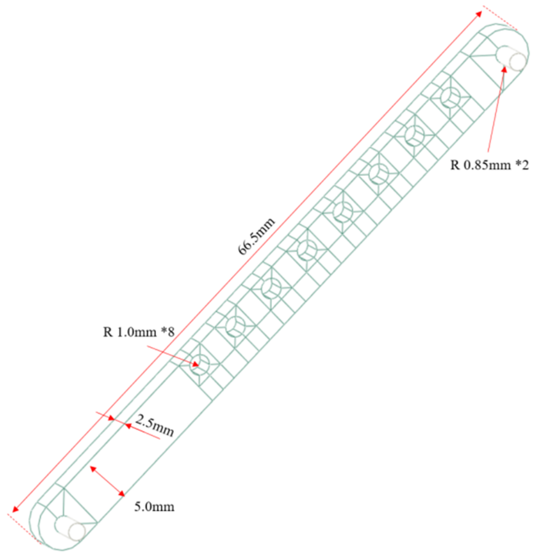

Figure S1: Finite element model of a metal coupler beam, where *2 and *8 indicate that there are two pin holes and eight stop holes in the model, respectively.

The model used an implicit static analysis step (Standard analysis in Abaqus), with a total of two analysis steps defined. The first step applies only a small displacement along the axial direction of the metal pins to enable the contact between the metal pin and the metal coupler beam, improving the convergence of the model; the second step removed the axial displacement boundary condition of the metal pin and applied a concentrated load on the metal pin.

Boundary conditions

The boundary conditions were defined by fixing one of the metal pins and applying a concentrated force in the cross-section of the other pin by means of Multipoint Control (MPC). In MPC, the constraint type was defined as binding, restricting the degree of freedom (DOF) of mesh nodes on both contacting surfaces. In addition, symmetric boundary conditions were defined on the cross-section of both metal pins, i.e., the displacement along the axial direction of the pin was constrained to be zero.

Mesh

Since the metal coupler beam had eight limit screw holes and two pin holes, the metal linkage structure needed to be geometrically partitioned for a good-quality meshing.

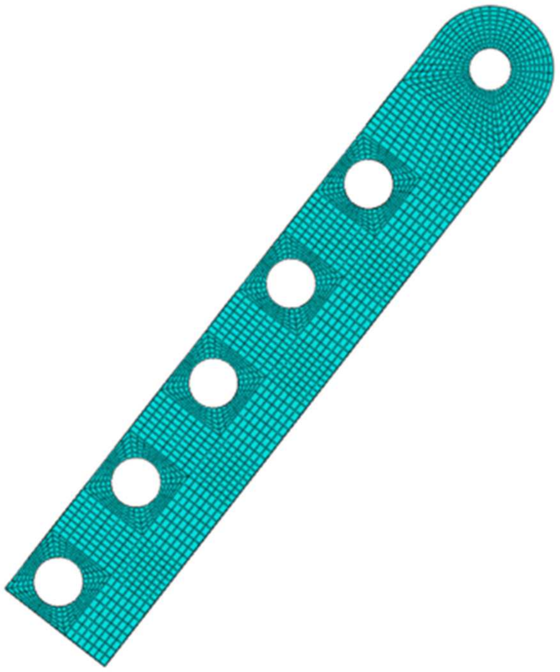

Figure S2: Local mesh distribution of screw holes

Various sizes of mesh were mapped around the stop holes to check the change of stress surrounding the hole. In Sup figure 3, when the cell size at the screw hole edge was 0.25mm, the stress change was less than 3%. Therefore, the mesh size in the FE model was set to 0.25mm

surrounding the hole, and the rest was set to 0.5mm. A total of 53,306 nodes and 44,560 C3D8 cells (hexahedral cells) were mapped. The average aspect ratio of the mesh was 1.18, the maximum aspect ratio was 1.72, and the minimum aspect ratio was 0.96. The average value of the in-plane angle of the hexahedral cells was 92.24, the maximum angle was 123.64°, and the minimum angle was 65.90°.

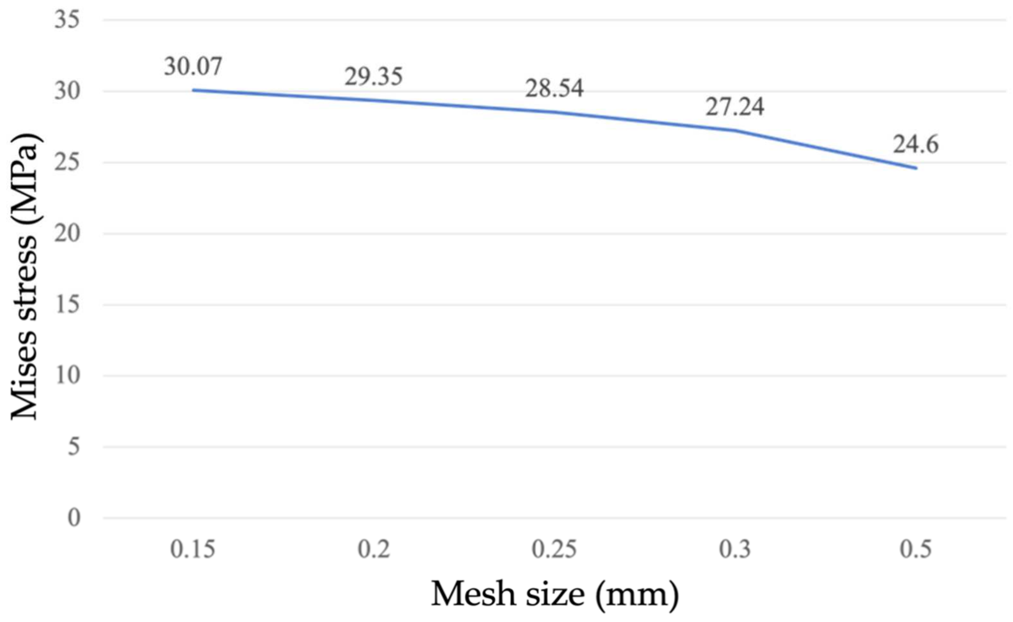

Figure S3: Trend of Mises stress changing with various mesh sizes.

Mises stress distribution in the metal coupler beam

The maximum Mises stress of the metal connecting rod structure was 146.8 MPa under 280N concentrated force load, and the maximum stress appeared around the pin hole. Due to the eccentric design of the stop hole, the Mises stress distribution showed an asymmetric pattern, and the stress surrounding stop holes was the largest (106.3MPa) on the side with smaller hole edge distance. The maximum Mises stress around the pin hole and the stop hole was much less than 0.2% yield strength of 7075 aluminum alloy, thus, the coupler beam cannot be plastically deformed or damaged.

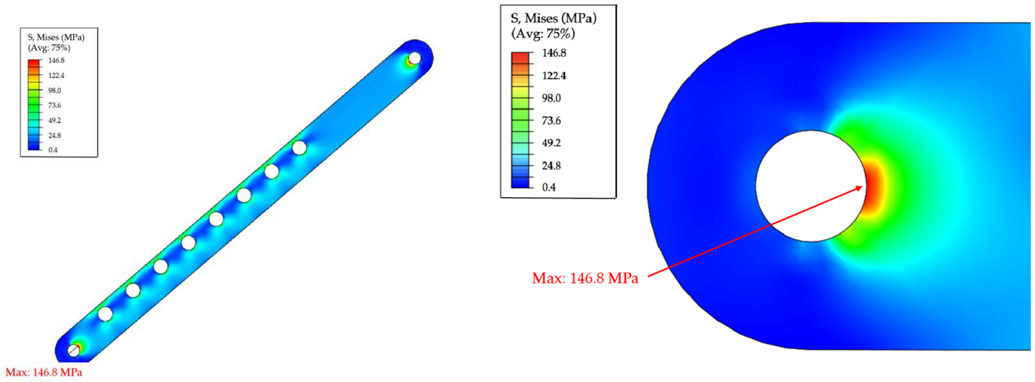

(**a**) (**b**)

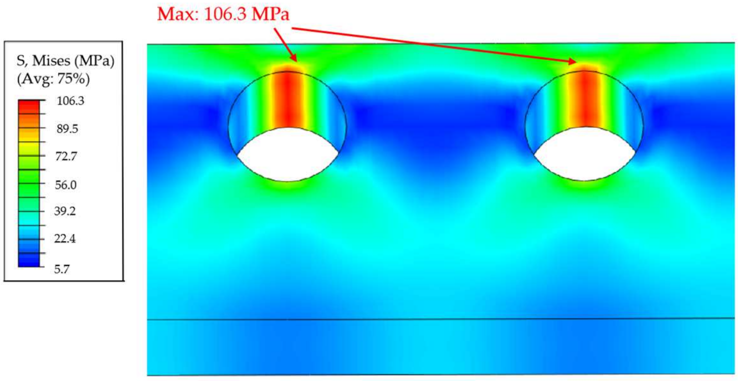

(**c**)

Figure S4: Mises stress distribution under a 280N compression (the stop nail and metal pins are hidden). **(a)** coupler bar; **(b)** pin hole; **(c)** stop holes
